# Supplementary material for: Comparison of CRISPR–Cas9 Tools for Transcriptional Repression and Gene Disruption in the BEVS
Source: Viruses. 2021 Sep 24;13(10):1925. doi: 10.3390/v13101925 (PMC8541072; doi:10.3390/v13101925)
Supplement: Supplementary file 1 [file viruses-13-01925-s001.zip › viruses-1326414-supplementary.pdf]

Supplementary Tables

Table S1. Primers used in this study.

Plasmid	Sequence (5′ – 3′)	Use (template)
pOpIE2-dCas9-puro	ctgtcattatcttagtttgtattgtcatg<br>gctcatgttgtgtcgctg	OpIE2 5'/3' UTR (pOpIE2E2.3)
	tacagcgacacaacatgagcatggacaagaagtattctatcggactg<br>ccctcaagcttgcctgatccacctaccttgcgcttcttcttg	dCas9 (pdCas9:BFP)
	ggatcaggcaagcttgagg<br>acaaactaagataatgacagctggatccctcgagtcagg	T2A/pac (pAc-sgRNA-Cas9)
pOpIE2-Cas9-puro	tacagcgacacaacatgagcatggacaagaagtattctatcggactg	Cas9-puro (pAc-sgRNA-Cas9)
rBEV transfer plasmids	cactaacctaggtagctgagcgc<br>cgctggactggcatgaac	rBEV ORF603/ORF1629
OpIE2GFP-sgRNA	cgaagttcatgccagtccagcggcaaggcgattaagttgggta<br>gctcatgttgtgtcgctg	OpIE2 5' UTR
	tacagcgacacaacatgagcatggtgagcgtgatcaagc<br>caaactaagataatgacagttacttggcctggctgggcagc	GFP CDS
	ctgtcattatcttagtttgtattgtcatg<br>gattaagttgggtaacgccagttc	OpIE2 3' UTR
	gaactggcgttacccaacttaatcaatctgcttagggttaggcg<br>ctcagctacctaggttagtggagagtgcaccatatgcggt	SfU6-sgRNA
pACUW51-p10	gttctagtggttggctacgtatactccgcgttggagtcttgtgtgcta<br>tgctttatttgtaaccattataagctgctcgctatacactcgcatggag	p10 region (AcMNPV genome)
	gcagcttataatggttacaaataaagc<br>ggagtatacgtagccaaccactagaac	pACUW51 backbone
p10GFP-sgRNA	cgaagttcatgccagtccagcggatgaagtggttcgcatcct<br>ggtgatggtgcatgattgtaaataaaatgtaatttacag	p10 5'UTR
	atgaatcgtttttaaaataacaaatcaattgttttataa<br>gatgattaagttgggtaacgccagttcgacatgataagatacattg	p10 3'UTR
	atcatgcaccatcaccaccatcatatggtgagtgtgattaaaccag<br>gttattttaaaaacgattcatggcgcgccttacttggcctggctgg	replace p10 ORF with *gfp* gene
	gaactggcgttacccaacttaatcatctgcttagggttaggcgttttg<br>ctcagctacctaggttagtggagagtgcaccatatgcggt	SfU6-sgRNA
p6.9GFP-sgRNA	ccgaagttcatgccagtccagcgaaattccgttttgcgacg<br>ccatatgatggtggtgatggtgcatgtttaaattgtgtaatttatg	p6.9 5' UTR to replace p10 5' UTR
Retarget sgRNAs	gttttagagctagaaatagcaagttaaaataagg<br>cggtggtcgagcacga	retarget sgRNA† (Cas9 handle)<br>retarget sgRNA† (SfU6)
qPCR primers	cgacgttgctttttgatcct<br>gcaacgacaagccatcagta	28S
	gacgatcgctaggcatttag<br>gcgtgttgcttgtgaaac	VP39
	gacaacagggagaagattgag<br>ggaggttcttatcgaagttagtc	(d)Cas9
	tctacgacatcaggttcgacgg<br>tccttcttggccttgtaggtgg	GFP

†: spacer sequence appended to 5′ end of sequence.

Supplementary Figures

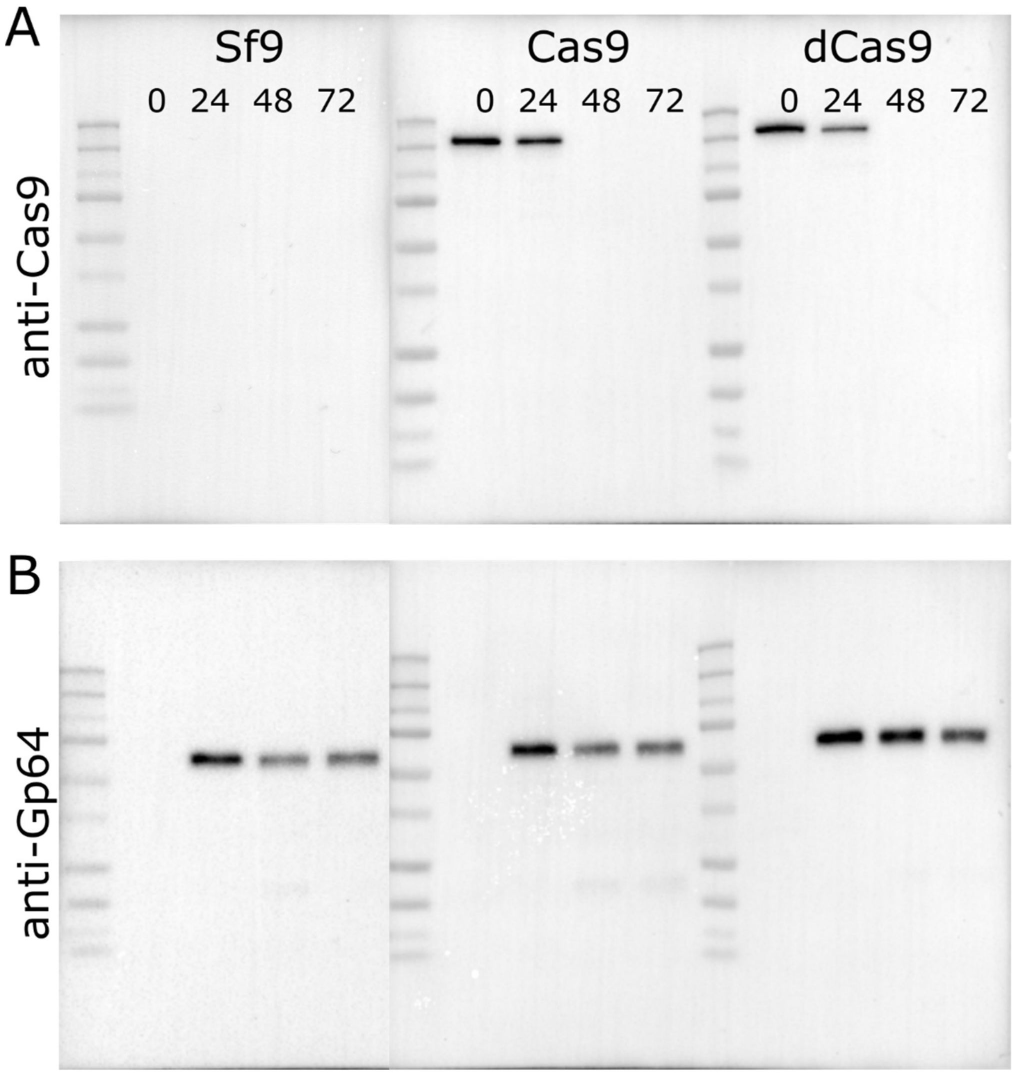

Figure S1. Expression of Cas9 and dCas9 is obstructed by infection. Western. blot analysis of infected Sf9, Sf9-Cas9, and Sf9-dCas9 cells for production of **A.** (d)Cas9 and **B.** GP64.

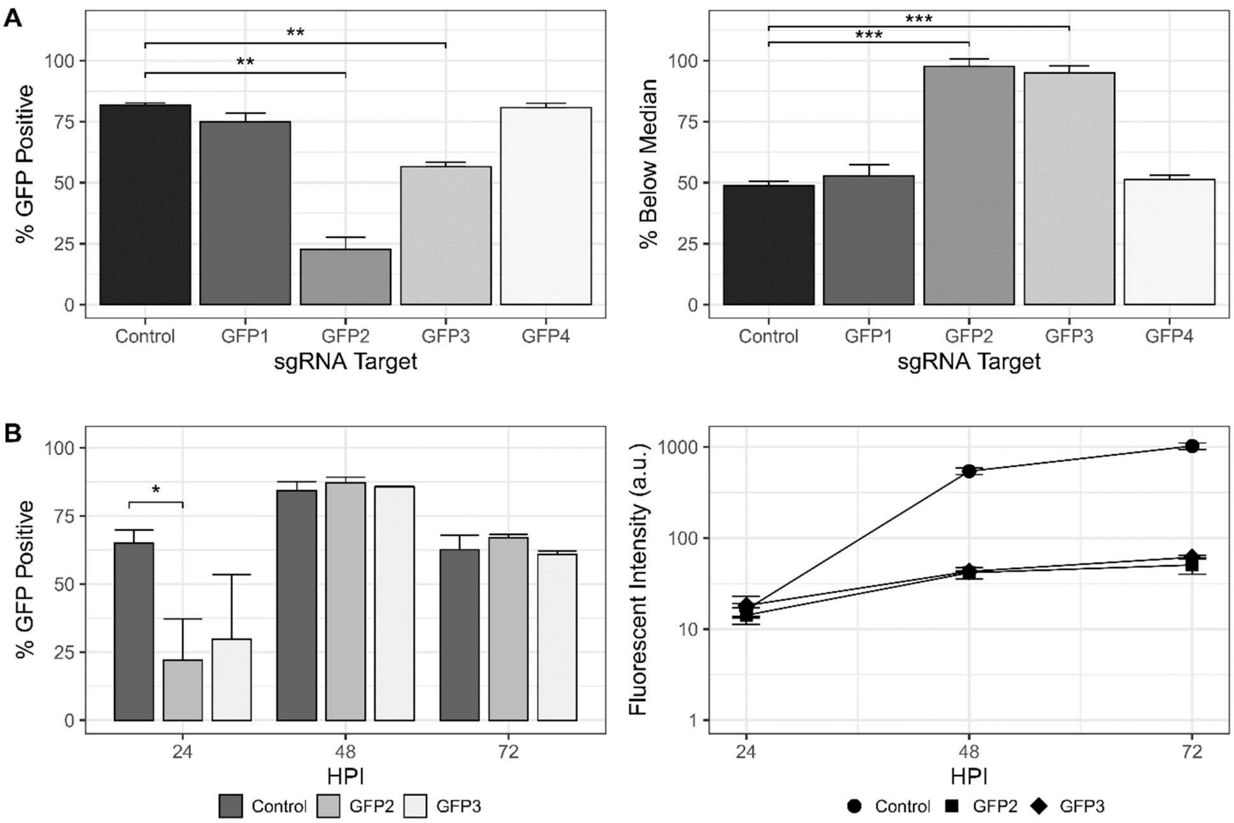

Figure S2. CRISPRi-mediated repression of *gfp* transcribed from OpIE2 or p10. promoters. A. Percent GFP-positive cells and proportion with fluorescence intensity below the median of the control for OpIE2GFP-sgRNA rBEVs and **B.** Percent GFP-positive and median fluorescence intensity for p10GFP-sgRNA rBEVs with sgRNAs targeting the *gfp* gene.

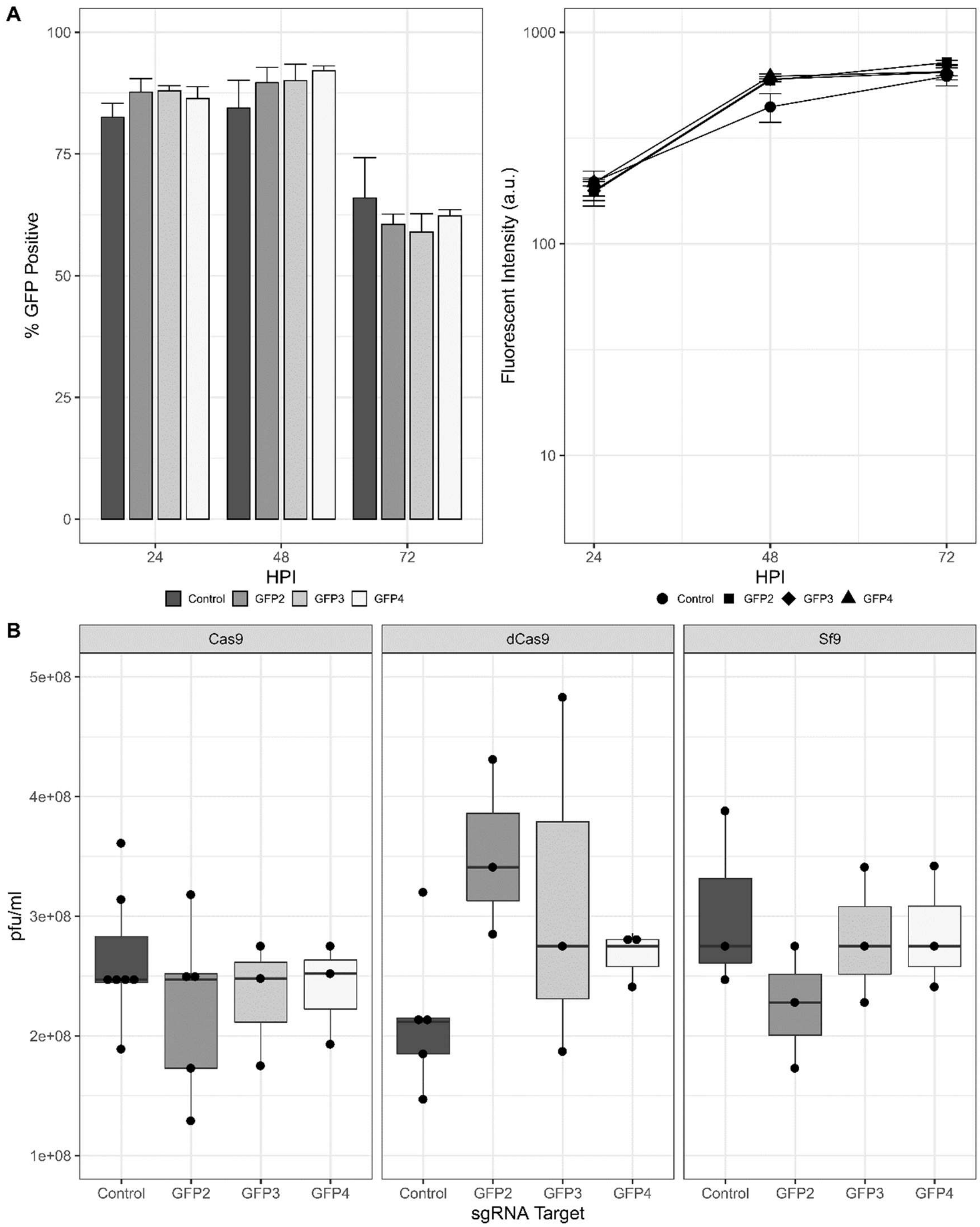

Figure S3. p6.9GFP-sgRNA rBEVs A. Control infections of p6.9GFP-sgRNA rBEVs. GFP2, GFP3, and GFP4 in Sf9 cells and **B.** IVT for GFP2, GFP3, and GFP4 from culture supernatants from infected Sf9-Cas9, Sf9-dCas9, and parental Sf9 cells.
